# Convallatoxin, a Dual Inducer of Autophagy and Apoptosis, Inhibits Angiogenesis *In Vitro* and *In Vivo*

**DOI:** 10.1371/journal.pone.0091094

**Published:** 2014-03-24

**Authors:** Seung Ya Yang, Nam Hee Kim, Yoon Sun Cho, Hukeun Lee, Ho Jeong Kwon

**Affiliations:** Department of Biotechnology, Translational Research Center for Protein Function Control, College of Life Science & Biotechnology, Yonsei University, Seoul, Korea; CHA University, Korea, Republic of

## Abstract

Autophagy and apoptosis are important processes that control cellular homeostasis and have been highlighted as promising targets for novel cancer therapies. Here, we identified convallatoxin (CNT), isolated from *Antiaris toxicaria*, as a dual inducer of autophagy and apoptosis. CNT exerts cytotoxic effects on a number of cancer and normal cell lines and induces apoptosis by increasing caspase-3 and poly ADP ribose polymerase (PARP) cleavage. Moreover, dose- and time-dependent autophagic activity was detected in CNT-treated cells, and mammalian target of rapamycin (mTOR)/p70S6K signal pathway inhibition was observed. Notably, CNT inhibits human umbilical vein endothelial cell (HUVEC) growth and exerts anti-angiogenic activity *in vitro* and *in vivo*. Collectively, these results demonstrate that the naturally occurring compound, CNT, is a novel anti-angiogenic compound via dual inducing of autophagy and apoptosis.

## Introduction

Programmed cell death (PCD) plays an essential role in embryonic development and cellular homeostasis. Defects in PCD contribute to diverse types of disease, including neurodegeneration, immune cell dysfunction, and cancer [1]. Particularly, cancer cells have evolved a variety of strategies to evade PCD to promote malignant growth. Thus, understanding the processes of PCD and designing therapeutic approaches to control cell death in cancer are considered important goals [2].

There are two major mechanisms of PCD: type I apoptotic cell death and type II autophagic cell death. Apoptosis is the most common form of PCD and is characterized by membrane blebbing, DNA fragmentation, and apoptotic body formation [3]. In contrast, autophagy is the major intracellular degradation pathway responsible for the removal of long-lived proteins or damaged organelles [4]. This process regulates cellular homeostasis for cell survival, but extensive autophagy induces cell death and bulk elimination of cells. Autophagic cell death is distinct from apoptosis; however, the relationship between autophagy and apoptosis is unclear, and it was recently reported that autophagy is concomitantly associated with apoptosis [5], [6].

Natural products have played significant roles in developing new bioactive small molecules and therapeutic agents for a number of diseases [7]. Several natural products act as anti-viral, anti-bacterial, and anti-cancer agents. For instance, vinblastine (Verban) was isolated from *Catharanthus roseus* (Apocynaceae) and was originally used for the treatment of diabetes as an oral hypoglycemic agent. Instead, it was found to be active against lymphocytic leukemia in mice. Semi-synthetic analogs of vinblastine are currently used in combination with other chemotherapeutic drugs for the treatment of cancers such as leukemias, lymphomas, and breast and lung cancers [8]. This example highlights how natural products can be used as tools for identifying novel natural small molecules with unique chemical structures and biological activities.

As part of our continuous efforts to identify new small molecules isolated from natural plants that induce cell death, we screened 3,000 crude natural plant extracts for their effects on HeLa cell growth by using cell-based screening. Herein, we found that convallatoxin (3-O-R-L-rhamnopyranosylstrophanthidin, CNT), a natural small molecule, induces cell death through a combination of apoptosis and autophagy. Notably, CNT exerts better inhibitory activity toward HUVEC proliferation and anti-angiogenic effects in HUVECs and in chorioallantoic membrane assays. CNT is isolated from the trunk bark of *Antiaris toxicaria*, which contains a complex mixture of cardiac glycosides [9], [10]. Cardiac glycosides are well known Na^+^/K^+^-ATPase inhibitors, and some of them are used to treat congestive heart failure and atrial arrhythmias. Recent studies have reported that cardiac glycosides have potential as anticancer agents, and CNT also exerts a cytotoxic effect on diverse cancer cell lines [11]–[14]. However, the detailed mechanisms underlying CNT-induced cell death and anti-angiogenic activity have not been explored. Here, we report for the first time that CNT is a novel natural small molecule that inhibits angiogenesis *in vitro* and *in vivo by* inducing cell death via dual induction of apoptosis and autophagy.

## Materials and Methods

### Materials

CNT was purchased from Sigma-Aldrich (St. Louis, MO, USA). Dulbecco's modified Eagle medium (DMEM), fetal bovine serum (FBS), Hoechst 33342, and LysoTracker Red were obtained from Invitrogen (Grand Island, NY, USA). Endothelial basal media-2 (EBM-2) was purchased from Cambrex Bio Science (Walkersville, MD, USA). Monodansylcadaverine (MDC) was obtained from BioChemika (Buchs, Switzerland). The caspase family inhibitor Z-VAD-FMK was from Promega (Madison, WI). Transwell plates, recombinant human vascular endothelial cell growth factor (VEGF), and Matrigel were obtained from Corning (Corning, NY), KOMA Biotech., Inc. (Seoul, Korea), and BD Biosciences (Bedford, MA), respectively. LC3, mammalian target of rapamycin (mTOR), phospho mTOR, p70S6K, phospho p70S6K, Atg5, poly ADP ribose polymerase (PARP), cleaved caspase-3, and tubulin antibodies were purchased from MBL (Nagoya, Japan), Cell Signaling Technology (Beverly, MA), and Millipore (Billerica, MA), respectively.

### Cell culture

HeLa (human cervical carcinoma), A549 (human lung carcinoma), HEK293 (human embryonic kidney 293), CHANG (human liver, purchased from ATCC) cells were grown and maintained in DMEM with 10% FBS and 1% antibiotics. HT1080 (human fibrosarcoma) and U87MG (human glioblastoma) cells were grown and maintained in MEM, and HepG2 (human liver carcinoma) cells were grown in RPMI 1640 with 10% FBS and 1% antibiotics. HUVECs were grown for 6–11 passages in EBM-2 medium supplemented with 10% FBS. Cells were cultured at 37°C in an atmosphere of 5% CO_2_ in air.

### Measurement of cell growth and viability

Cell proliferation was measured with a 3-(4,5-dimehylthiazol-2-yl)-2,5-diphenyl tetrazolium bromide (MTT, Sigma-Aldrich) colorimetric assay. Various cell lines were seeded in 96-well plates and incubated overnight. Cells were treated with various concentrations of CNT for 3 days, and 2 mg/mL MTT was added to each well and incubated for 3–4 h. MTT formazan was dissolved in dimethyl sulfoxide (DMSO), and the absorbance was read at 540 nm with microplate reader (Bio-Tek Instrument Inc., Winooski, VT). Viability assay was assessed using trypan blue staining [15].

### DNA content analysis

Cells treated with CNT were harvested and fixed in 70% ethanol on ice for 30 min. After centrifugation (2,000 rpm), the cell pellets were washed once with phosphate-buffered saline (PBS) and incubated with propidium iodide (PI, 50 µg/mL) and RNase A (100 µg/mL) at 37°C. After 1 h, the DNA contents of 10,000 events were measured with fluorescence-activated cell sorting (FACS) on a FACScan (Becton Dickinson, San Jose, CA). Data were analyzed by Cell Quest software (Becton Dickinson).

### DNA fragmentation assay

For detection of DNA fragmentation, cells were collected and washed with PBS. Then they were lysed in 0.3 M Tris-HCl (pH 7.5) containing 0.01 M ethylenediaminetetraacetic acid (EDTA), 0.1 M NaCl, and 0.2 M sucrose. Then lysates were incubated with 10% sodium dodecyl sulfate (SDS) at 65°C for 30 min, then 5 M potassium acetate was added to lysates on ice, and supernatants were collected after centrifugation at 13,000 rpm for 10 min. Next, the supernatant was incubated with 20 µg/mL RNase A for 1 h, and phenol/chloroform extraction and ethanol precipitation were performed to extract the DNA. After electrophoresis for 1 h at 50 V in a 1.2% agarose gel, DNA was visualized by staining with ethidium bromide.

### Caspase inhibitor assay

Cells were seeded in a 12-well plate and incubated overnight at 37°C. They were pretreated with Z-VAD-FMK (caspase family inhibitor) or DMSO for 1 h before treatment with CNT or camptothecin for 24 h. Cell viability was determined by trypan blue staining.

### Visualization and analysis of intracellular autophagic vacuoles

To detect autophagic vacuoles, monodansylcadaverine (MDC), a fluorescent dye known as specific marker for autophagic organelles, was treated to the cells at 50 µM for 30 min (15). After incubation, the cells were washed three times with PBS and immediately analyzed by fluorescence microscopy. Fluorescence intensity was quantitatively measured with ImageJ v1.43 (Bethesda, MD) and is expressed as arbitrary units. LysoTracker Red, a fluorescent dye for labeling functional lysosomes [16], was applied to the cells at 50 nM for 30 min with Hoechst 33342 (1∶1,000). After washing, cells were visualized by fluorescence microscopy or analyzed using ArrayScan VTI HCS Reader (Cellomics, Inc., Pittsburgh, PA). The target activation protocol in the HCS Reader was used to identify individual cells by their Hoechst-labeled nuclei and to analyze changes of LysoTracker Red fluorescence within each cell.

### Western blot analysis

The cell lysates were separated by 8–12.5% SDS-polyacrylamide gel electrophoresis (SDS-PAGE) and transferred to polyvinylidene fluoride membranes (Millipore) using standard electroblotting procedures. Blots were then blocked and immunolabeled overnight at 4°C with primary antibodies. Immunolabeling was detected by an enhanced chemiluminescence kit (GE Healthcare, Buckinghamshire, UK) according to the manufacturer's instructions.

### *In vitro* capillary tube formation assay

Matrigel was coated in a 48-well plate and polymerized for 2 h at 37°C. HUVECs (5×10^4^ cells/well) were seeded on the Matrigel surface and treated with VEGF (30 ng/mL). Then, compound was added and incubated for 3–18 h at 37°C. Cellular morphological changes and tube formations were observed with a microscope (IX71, Olympus, Tokyo, Japan) and photographed (DP70, Olympus) [16].

### *In vitro* chemoinvasion assay

HUVEC invasiveness was examined *in vitro* using a Transwell chamber system with 8.0-µm pore-size polycarbonate filter inserts. The bottom of the filter was coated with 10 µL gelatin (1 mg/mL), and the top was coated with 10 µL Matrigel (3 mg/mL). CNT was added to the lower chamber in the presence of VEGF (30 ng/mL), and HUVECs (6×10^4^ cells/well) were placed in the top of the filter. After incubation for 18 h at 37°C, the cells were fixed with 70% methanol and stained with hematoxylin and eosin. Cell invasiveness was determined by counting the total number of invaded cells in the bottom of the filter using an Olympus IX70 microscope at 100× magnification [17]. Conditioned medium (CM) from Atg5 knocked-down cells were obtained from HeLa cells. Atg5 was knocked-down in HeLa cells by using siRNA targeting Atg5 gene (siAtg5) (On-TARGETplus Human ATG5 SMARTPOOL, Thermo Scientific). 20 nM siAtg5 was transfected into HeLa cells using Lipofectamine 2000 (Invitrogen), according to the manufacturer's protocol. After 16 h of transfection, media was changed to serum free DMEM, and treated with CNT or DMSO for 12 h. Media was then obtained and concentrated by Amicon Ultra centrifugal filter 3K membrane (Millipore, Ireland) according to the manufacturer's protocol, and media was exchanged to EBM-2 for *in vitro* invasion assay. 20 µM Z-VAD-FMK was pretreated 1 h before CNT treatment for 12 h. Then CM was obtained and exchanged to EBM-2 as mentioned previously.

### Chick chorioallantoic membrane (CAM) assay

The CAM assay was conducted as previously described [18]. Briefly, fertilized chicken eggs were kept in a humidified incubator at 37°C for 3 days. Approximately 4–5 mL egg albumin was removed with a hypodermic needle after allowing the CAM and yolk sac to drop away from the shell membrane. On day 5, a 2.5-cm diameter window was made with a razor and tweezers, and compound-loaded Thermanox coverslips (NUNC, Rochester, NY) were placed on the CAM surface. Two days later, 2–3 mL Intralipose (Green Cross Co., Yongin, Korea) was injected beneath the CAM, and the membrane was observed under a microscope. Retinoic acid (RA) was used as a positive control.

### Statistical analysis

Results were expressed as means ± standard error (SE). Student's t-tests were used to determine the statistical significance between control and test groups. *P*<0.05 was considered statistically significant.

## Results

### Crude extract of *Antiaris toxicaria* and CNT inhibit cell growth

**Figure 1:**
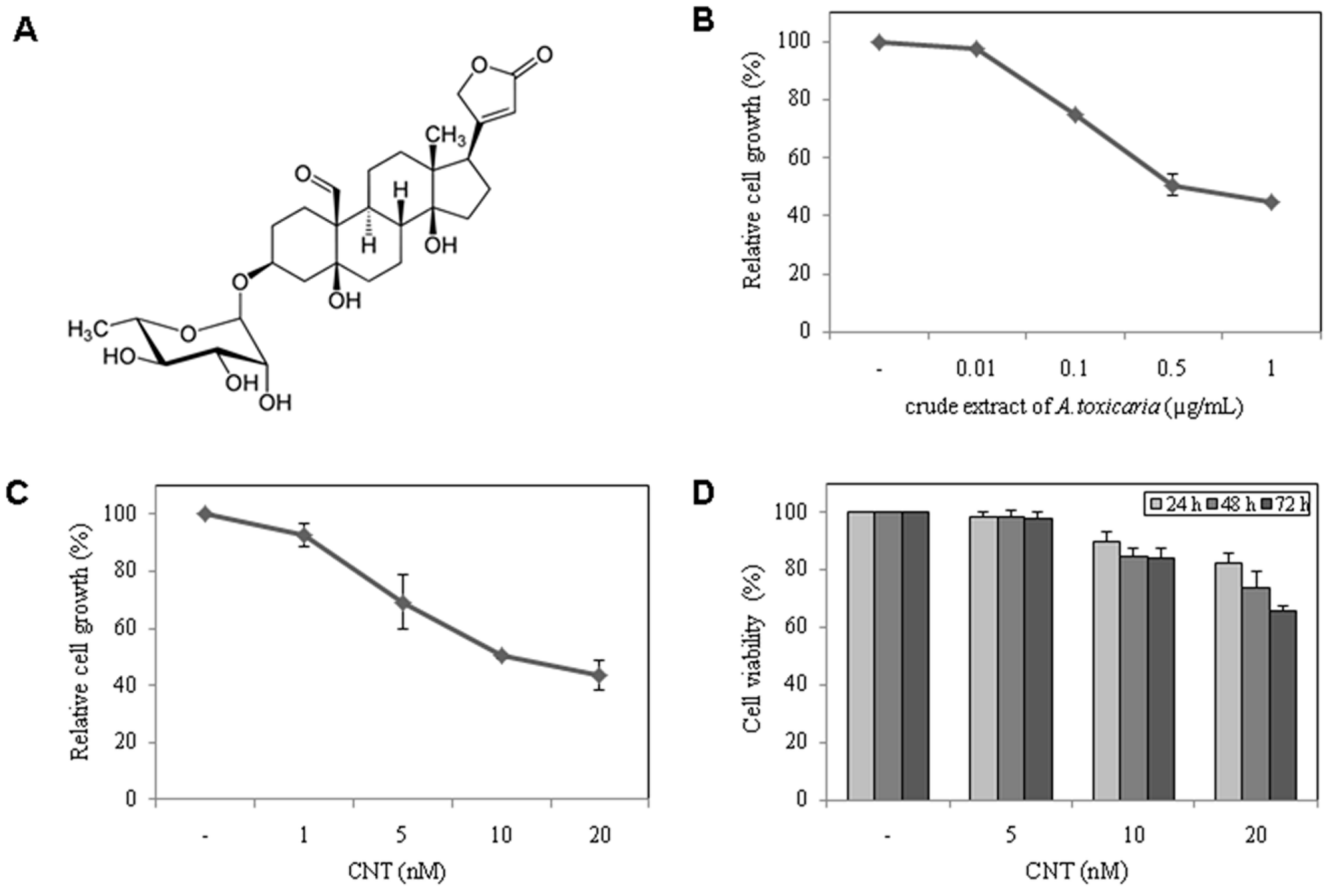
Chemical structure and cytotoxic effect of CNT on HeLa cells. (A) The chemical structure of CNT (C_29_H_42_O_10_, MW 550.64). (B) The effect of crude extract of *Antiaris toxicaria* on cell growth. (C) The effect of CNT on cell growth. (D) The effect of CNT on cell viability.

To identify novel naturally occurring small molecules that inhibit cell growth, we first treated HeLa cells with 3,000 natural plants extracts. We found that *Antiaris toxicaria* crude extract dose-dependently inhibited HeLa cells growth (Fig. 1B). CNT is a known principal component of this extract (Fig. 1A); therefore, we investigated whether CNT was responsible for the observed inhibition of cell growth. As shown in Fig. 1C, CNT potently inhibited cell growth with a half maximal inhibitory concentration (IC_50_) of 14 nM, and the cytotoxic effect on HeLa cells was dose and time dependent (Fig. 1D). CNT also inhibited growth in a variety of other cancer and normal cell lines (Table 1). These results demonstrate that the natural small molecule CNT has strong cell death-inducing activity.

**Table 1:** IC_50_ values of CNT on various cell lines.

	Cancer cells					Normal cells		
Cell lines	HeLa	U87MG	HepG2	HT1080	A549	HUVECs	HEK293	CHANG
IC_50_ (nM)	14	16	40	34	18.5	4	14	16

### CNT induces apoptotic cell death

**Figure 2:**
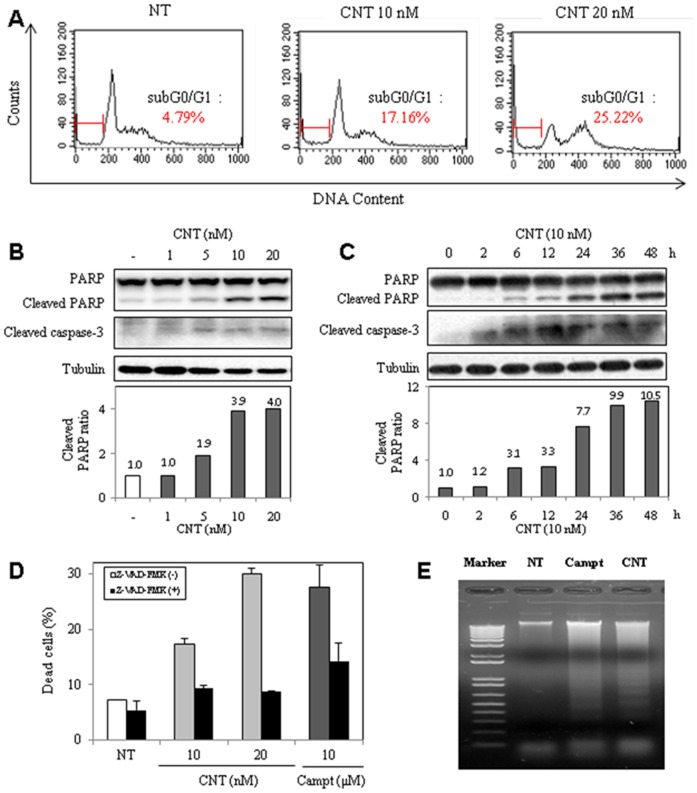
CNT-induced apoptosis in HeLa cells. (A) Enhancement of sub G0/G1 cell population by CNT for 2 days, and analyzed for apoptosis by FACS. (B) Increased cleavage of PARP and caspase-3, in a dose-dependent manner after 24 h. (C) Increased cleavage of PARP and caspase-3 in a time-dependent manner with 10 nM CNT. Tubulin was used as an internal control. (D) Effect of apoptosis inhibitor on CNT-treated HeLa cells. (E) Detection of DNA fragmentation in CNT- or camptothecin-treated cells.

To investigate whether CNT-mediated growth inhibition was due to apoptosis, DNA content analysis was performed on HeLa cells (Fig. 2A). CNT enhanced the sub-G0/G1 population in HeLa cells in a dose-dependent manner. In addition, cleavage of caspase-3 and PARP, which are considered hallmarks of apoptosis, were investigated by western blot analysis (Fig. 2B, C), which demonstrated that caspase-3 and PARP were cleaved in CNT-treated cells in dose- and time-dependent manners. After pretreatment with caspase-3 and apoptosis inhibitor Z-VAD-FMK, trypan blue assays were performed to assess the viability of CNT-treated cells (Fig. 2D). The number of dead cells decreased in the CNT- and camptothecin (known as apoptosis inducer)-treated cells that were also treated with Z-VAD-FMK. Fragmented DNA was also detected in CNT-treated cells, similar to what was observed in camptothecin-treated cells (Fig. 2E). Accordingly, the results suggest that growth inhibition in CNT-treated HeLa cells is partly due to induction of apoptosis.

### CNT induces autophagy through the mTOR signaling pathway

**Figure 3:**
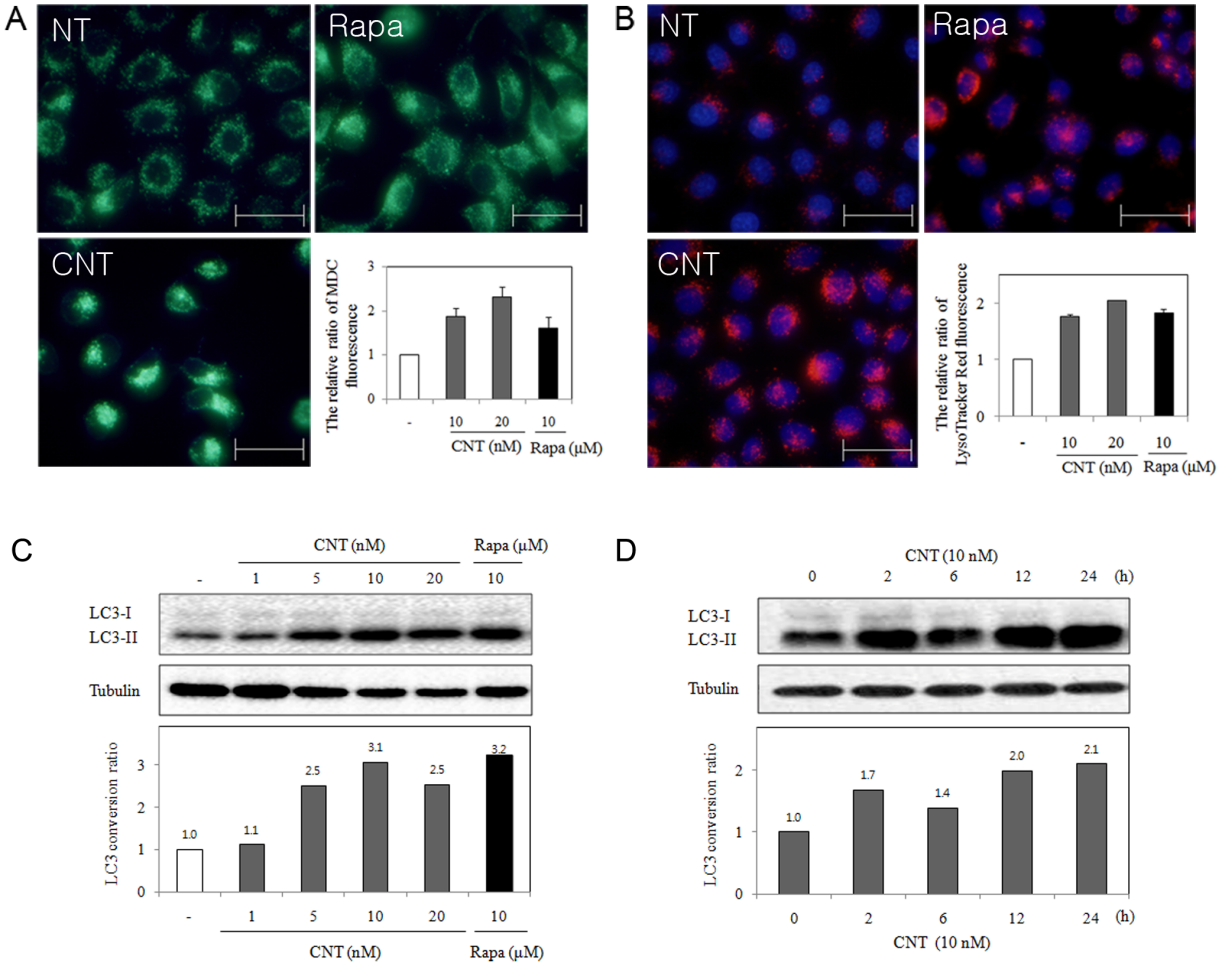
Autophagic activity of CNT. (A) Detection of autophagic vacuoles by MDC staining in HeLa cells incubated for 24 h in the absence or presence of 10 nM CNT or 10 µM rapamycin (N: non-treatment, Rapa: rapamycin, CNT: convallatoxin). (B) Detection of CNT-induced autophagic vacuoles by LysoTracker Red. HeLa cells were treated with 10 nM CNT or 10 µM rapamycin for 24 h. Scale bars indicate 50 µm. (C) The conversion of LC3-I to LC3-II in CNT-treated HeLa cells after 24 h with different concentrations of CNT or 10 µM rapamycin. (D) The conversion of LC3-I to LC3-II following treatment with 10 nM CNT over time. Tubulin was used as an internal control.

We next investigated the autophagic activity of CNT. Cells were stained with MDC and LysoTracker Red, fluorescent dyes known as specific markers for autophagic organelles. After treatment with CNT for 24 h, increased MDC (Fig. 3A) and LysoTracker Red (Fig. 3B) fluorescence intensities were observed in HeLa cells whereas there was no staining of MDC to nucleus and the morphology of nucleus was not changed with CNT treatment (Fig. S1). Conversion of cytosolic microtubule-associated protein I light chain 3 (LC3) I to autophagosome-associated LC3-II, a well-known marker of autophagosome assembly, was assessed after CNT treatment. LC3 conversion was remarkably enhanced in cells treated with 24 h with a wide range of CNT concentrations (Fig. 3C). HeLa cells treated with 10 nM of CNT exhibited increased LC3-II levels in time-dependent manner (Fig. 3D). Collectively, these results demonstrate that CNT induces both autophagy and apoptosis in HeLa cells.

**Figure 4:**
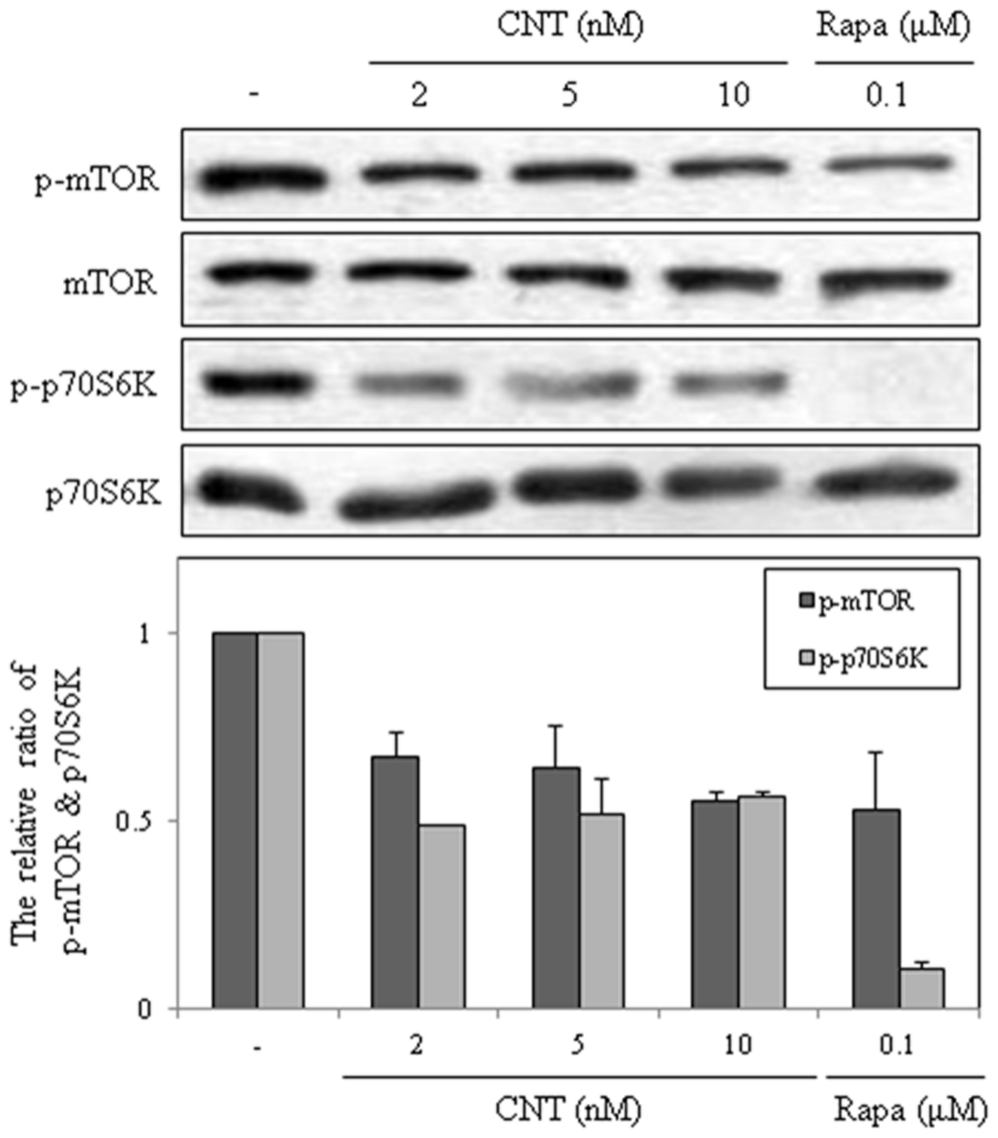
Effect of CNT on the mTOR/p70S6K signaling pathway. Tubulin was used as an internal control.

A well-known autophagy signal pathway is mTOR, which integrates various upstream signal transduction pathways and negatively controls autophagy. Rapamycin activates autophagy by inhibiting mTOR signaling. Therefore, the level of mTOR was investigated in CNT-treated cells. Similar to rapamycin, CNT inhibited mTOR signaling in HeLa cells, and p70S6K, a substrate of mTOR, was markedly decreased (Fig. 4). These results indicate that mTOR signaling is involved in CNT-induced autophagy.

### CNT inhibits angiogenesis *in vitro* and *in vivo*

**Figure 5:**
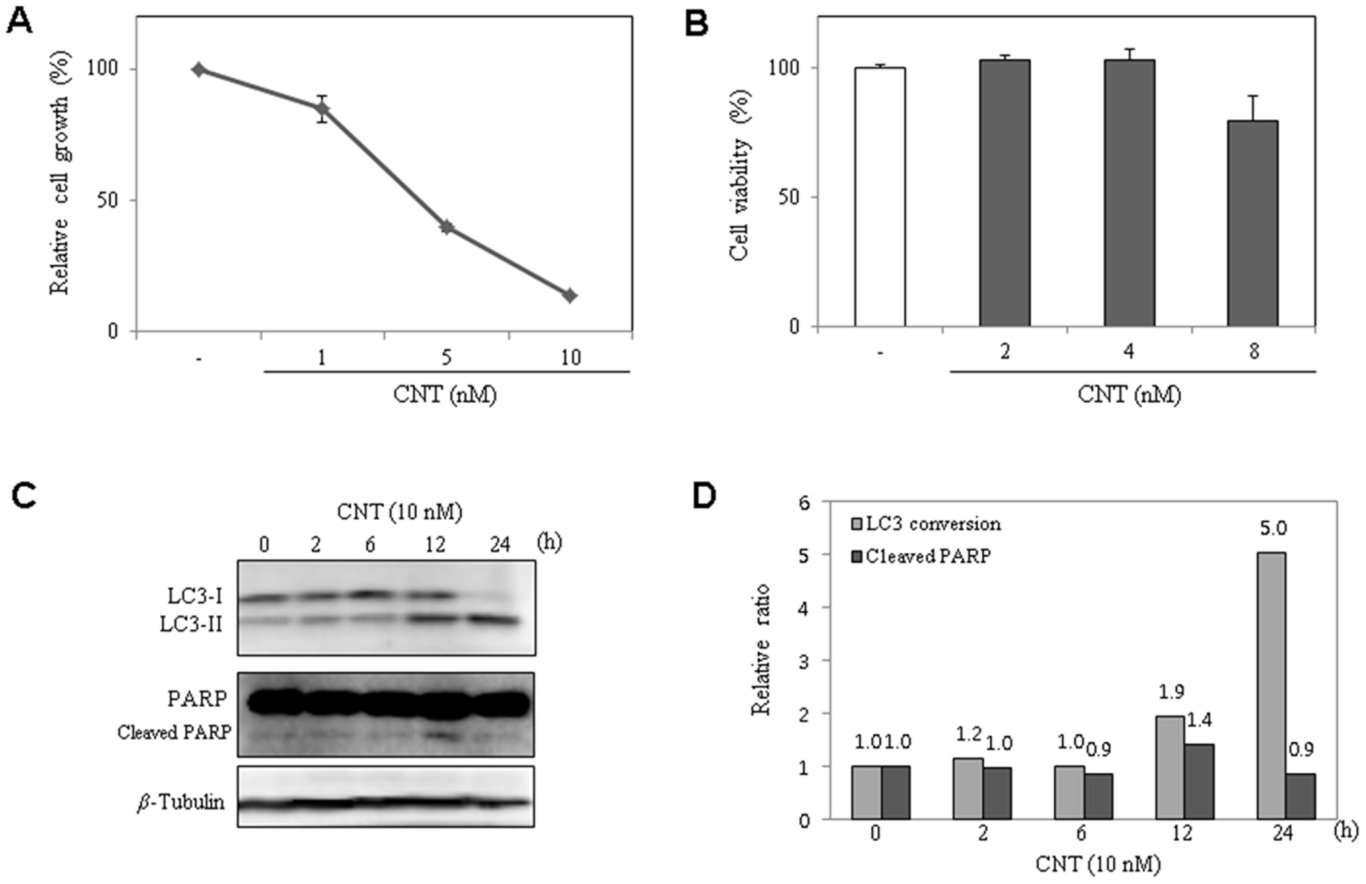
Anti-proliferative activity of CNT on HUVECs. (A) Growth inhibitory activity of CNT on HUVECs. (B) The effect of CNT on HUVEC viability as determined by the trypan blue assay. (C) The conversion of LC3-I to LC3-II and increased cleavage of PARP following treatment with 10 nM CNT over time on HUVECs. Tubulin was used as an internal control. (D) Relative ratio of LC3 conversion and cleaved PARP was calculated as the percentage of LC3-I conversion to LC3-II and PARP to cleaved PARP, respectively.

**Figure 6:**
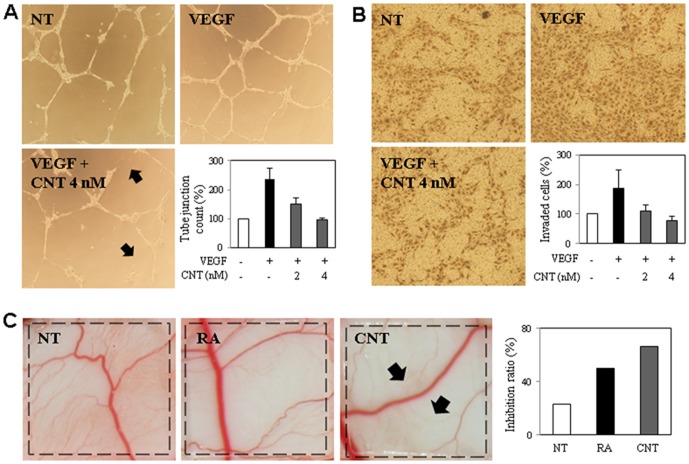
Anti-angiogenic activity of CNT *in vitro* and *in vivo*. (A) Effect of CNT on HUVEC tube-forming ability. Arrows indicate broken tubes formed by VEGF-stimulated HUVECs. (B) Inhibitory activity of CNT on HUVEC invasiveness. (C) Effect of CNT on *in vivo* angiogenesis assay, CAM (NT: EtOH control, RA: Retinoic acid (1 µg/egg), and CNT (0.5 ng/egg)). Arrows indicate compound-mediated inhibition of CAM neovascularization. The inhibition ratio was calculated as the percentage of inhibited eggs relative to the total number of eggs tested.

Angiogenesis is the process of new blood vessel formation from pre-existing ones and plays a critical role in diverse diseases, including solid tumor growth and metastasis [19]. Accordingly, angiogenesis inhibition has been highlighted as promising strategy for treating cancer. Notably, CNT exhibited a stronger growth inhibition effect on HUVECs compared to the other cell lines tested (Table 1 and Fig. 5A). Accordingly, the effect of CNT on cell viability was examined using a trypan blue assay. As shown in Fig. 5B, CNT-treated HUVECs exhibited no cytotoxicity at concentrations up to 4 nM for 3 days. However, CNT induced both autophagy and apoptosis on HUVECs by detection of LC3 conversion and PARP cleavage (Fig. 5C, D) at the concentration 10 nM. Accordingly, we investigated the effect of CNT on HUVEC angiogenic phenotypes at a dose of 2–4 nM with various angiogenesis *in vitro* and *in vivo* assays. First, a tube formation assay was assessed in which serum-starved HUVECs were stimulated by VEGF with or without concurrent CNT treatment. CNT inhibited VEGF-induced HUVEC tube formation in a dose-dependent manner (Fig. 6A). The effect of CNT on VEGF-induced HUVEC invasive activity was also investigated, and the results showed that CNT dose-dependently inhibited HUVEC invasiveness (Fig. 6B). These data indicate that CNT effectively inhibits VEGF-induced angiogenesis *in vitro*.

Next, the effect of CNT on blood vessel formation was analyzed *in vivo* using the CAM assay. After treatment with CNT for 2 days, the CAM was observed under a microscope. Normally, developed CAMs exhibit extensive capillary networks. However, CNT-treated CAMs showed the inhibition of capillary formation with no signs of thrombosis or hemorrhage (Fig. 6C). Collectively, these results demonstrate that CNT potently inhibits angiogenesis both *in vitro* and *in vivo*.

### Inhibition of autophagy and apoptosis inhibited CNT-induced effect on angiogenesis

**Figure 7:**
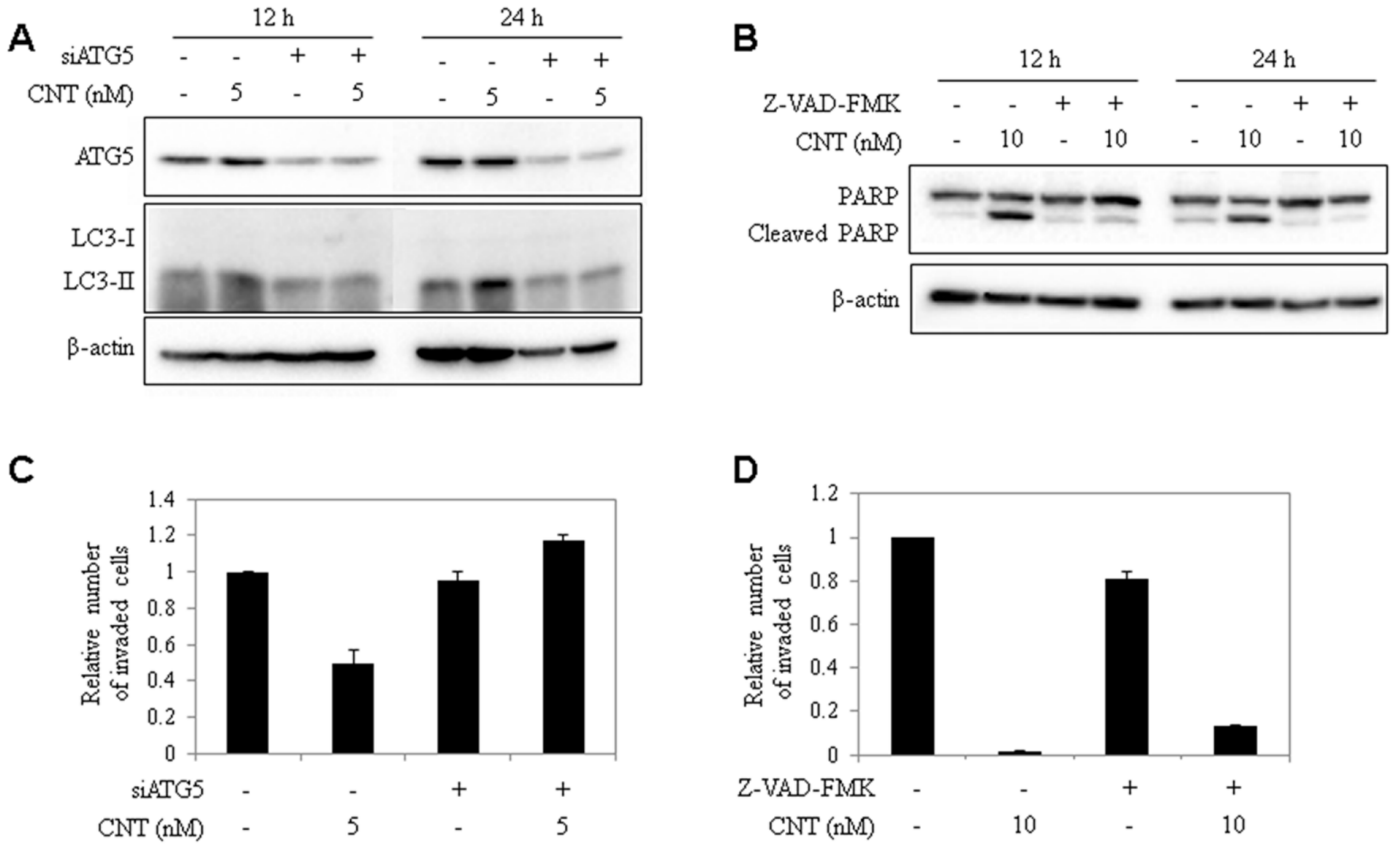
Anti-angiogenic effect of CNT decreased in autophagy- or apoptosis-inhibited cells. (A) Atg5 and LC3 conversion levels with 5 nM CNT treatment in normal HeLa cells or Atg5-knocked down cells. (B) Cleaved PARP levels with 10 nM CNT treatment in normal HeLa cells or Z-VAD-FMK pretreated cells. β-actin was used as an internal control. (C) Quantitative analysis of *in vitro* chemoinvasion assay with conditioned media from 5 nM CNT-treated HeLa cells or CNT-treated in Atg5-knocked down HeLa cells. (D) Quantitative analysis of *in vitro* chemoinvasion assay with conditioned media from 10 nM CNT-treated HeLa cells or with Z-VAD-FMK pretreated cells.

To address whether CNT effect on autophagy and apoptosis induction is responsible for angiogenesis inhibition of the compound, inhibition of autophagy and apoptosis was conducted by knockdown of Atg5 gene and caspase inhibition, respectively. First, 5 nM CNT was treated to examine the effect of CNT-induced autophagy. Autophagy inhibition by knockdown of Atg5 using siAtg5, resulted in inhibition of LC3 conversion induced by CNT at both 12 and 24 h (Fig. 7A). Secondly, 10 nM CNT was treated to examine the effect of apoptosis. Inhibition of apoptosis with Z-VAD-FMK inhibited PARP cleavage induced by CNT at both 12 and 24 h (Fig. 7B). Furthermore, *in vitro* chemoinvasion assay was assessed with conditioned-media (CM) obtained from autophagy or apoptosis inhibited HeLa cells, treated with CNT for 12 h to determine the effect of CNT on angiogenesis. CM from 5 nM CNT-treated cells inhibited invasion, but CM from CNT treatment in autophagy inhibited cells recovered the number of invaded HUVECs (Fig. 7C). Likewise, CM from 10 nM CNT-treated cells significantly inhibited invasion of HUVECs, but CM from apoptosis inhibited and CNT-treated cells recovered the number of invaded HUVECs slightly to 13% (Fig. 7D). The recovery of invaded cells in 10 nM CNT treatment in apoptosis inhibited cells may be lower than 5 nM CNT treatment in autophagy inhibited cells, probably due to the remaining autophagy activity of CNT (at 10 nM, CNT induces both autophagy and apoptosis). These results support that CNT inhibits angiogenesis by inducing autophagy and apoptosis.

## Discussion

Many cancer cells are resistant to existing anti-cancer agents because they are capable of evading cell death pathways. Therefore, the identification and characterization of new anti-cancer drugs that induce cell death via new mechanisms offer promising treatment for cancer. In this study, CNT exerted cytotoxic effects on diverse cancer cell lines by inducing both autophagy and apoptosis. Notably, all cells tested in this study showed potent sensitivity to CNT in the nanomolar ranges. CNT induced autophagy at 5 nM in HeLa cells; autophagic vacuoles and conversion of LC3 were detected. Also, CNT suppressed mTOR/p70S6K signaling starting from 2 nM, which shows similar effects compared to rapamycin. However, starting from 10 nM, both autophagy and apoptosis were induced. CNT induced apoptosis in HeLa cells by increasing caspase-3 and PARP cleavage along with LC3 conversion. These results demonstrate that CNT induces cell death via dual induction of autophagy and apoptosis. Accordingly, CNT would be a unique chemical tool to explore the underlying mechanism of autophagy and apoptosis in cell death. It is noteworthy that CNT exhibits stronger growth inhibitory activity on HUVECs than cancer cells, and the compound's anti-angiogenic activity was validated by various angiogenesis assays. Indeed, CNT exerted anti-angiogenic effects in VEGF-treated HUVECs cell assays *in vitro* and CAM assay *in vivo*. Also, anti-angiogenic effect of CNT decreased when autophagy or apoptosis were inhibited in cells. These results are in accordance with previous reports that of reduced VEGF levels in CNT-treated human glioma cells [20]. Nguyen *et al.* reported that the angiogenesis inhibitor, Kringle5, induces both autophagy and apoptosis in endothelial cells [21]. In addition, a recent study reports that paternally expressed gene 3 (Peg3), playing an important role in the p53/c-myc-mediated apoptosis pathway, is related with autophagy and angiogenesis pathway in endothelial cells [22]. Accordingly, our result is consistent with previous observations and demonstrates that CNT could exhibit anti-angiogenic activity via dual induction of autophagy and apoptosis. The anti-angiogenic activity of CNT is similar to rapamycin in that it induces autophagy through mTOR signaling and inhibits angiogenesis [25]. CNT is classified as a cardiac glycoside, a class of compounds that has a long history of therapeutic applications for heart disorder and cancer diseases [23]. Cardiac glycosides, including CNT, share a common structural motif of a steroid core with an unsaturated lactone ring and a sugar moiety. Na+/K+ ATPase is known as the binding target of cardiac glycoside including CNT. Na^+^/K^+^ ATPase plays key roles in regulating the diverse cell signaling events including autophagy and apoptosis by binding to cardiac glycosides [24]. Therefore, CNT induced autophagy and apoptosis may be functionally related with Na^+^/K^+^ ATPase signaling. Detailed intracellular mechanism underlying this possibility is under investigation.

**Figure 8:**
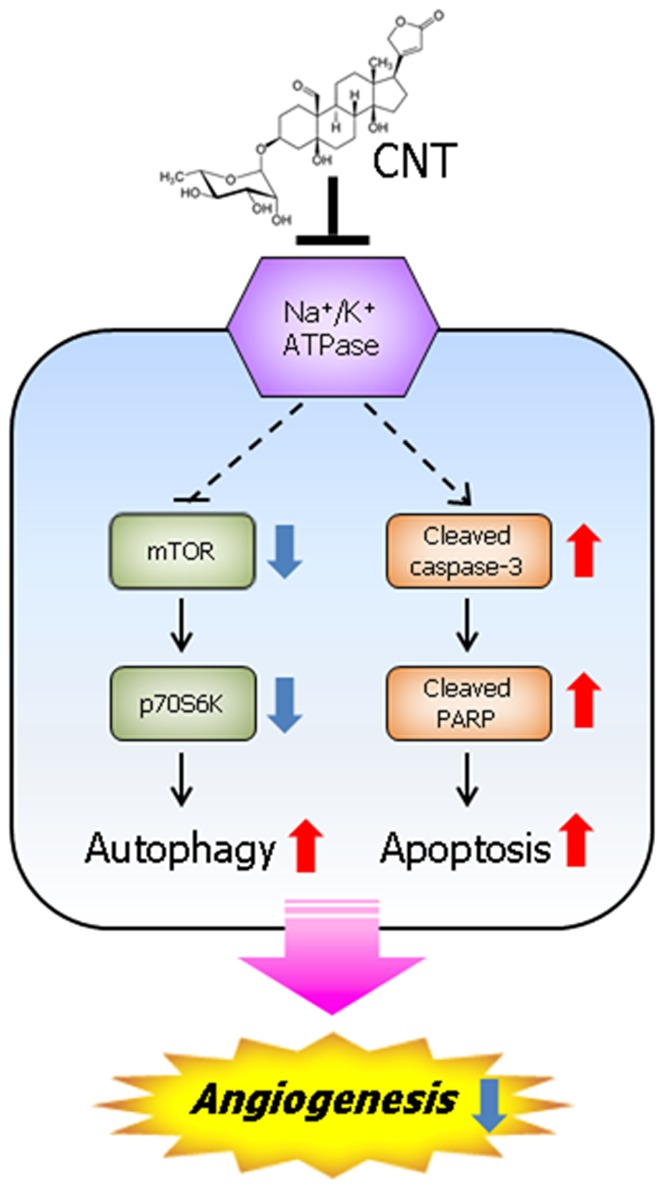
Schematic summary of CNT mediated signaling pathway related with anti-angiogenic activity.

Collectively, our results demonstrate that CNT, a dual inducer of autophagy and apoptosis for cell death, inhibits angiogenesis *in vitro* and *in vivo*. This compound could be used as a new chemical probe to explore the role of Na^+^/K^+^ ATPase in autophagy- and apoptosis-induced cell death and provide the basis of developing a new therapeutic agent for treating cancer or angiogenesis-related diseases (Fig. 8).

## Supporting Information

Figure S1 MDC stains autophagosomes in the cytosol but does not stain nuclei in HeLa cells. The vacuoles were monitored under fluorescence field and morphology of cell and nuclei were done under bright field (NT: non-treatment, Rapa: rapamycin, CNT: convallatoxin). Scale bar indicates 25 µm.
(TIF)
