# Supplementary figures and images for: Convallatoxin, a Dual Inducer of Autophagy and Apoptosis, Inhibits Angiogenesis In Vitro and In Vivo
Source: PLoS One. 2014 Mar 24;9(3):e91094. doi: 10.1371/journal.pone.0091094 (PMC3963847; doi:10.1371/journal.pone.0091094)

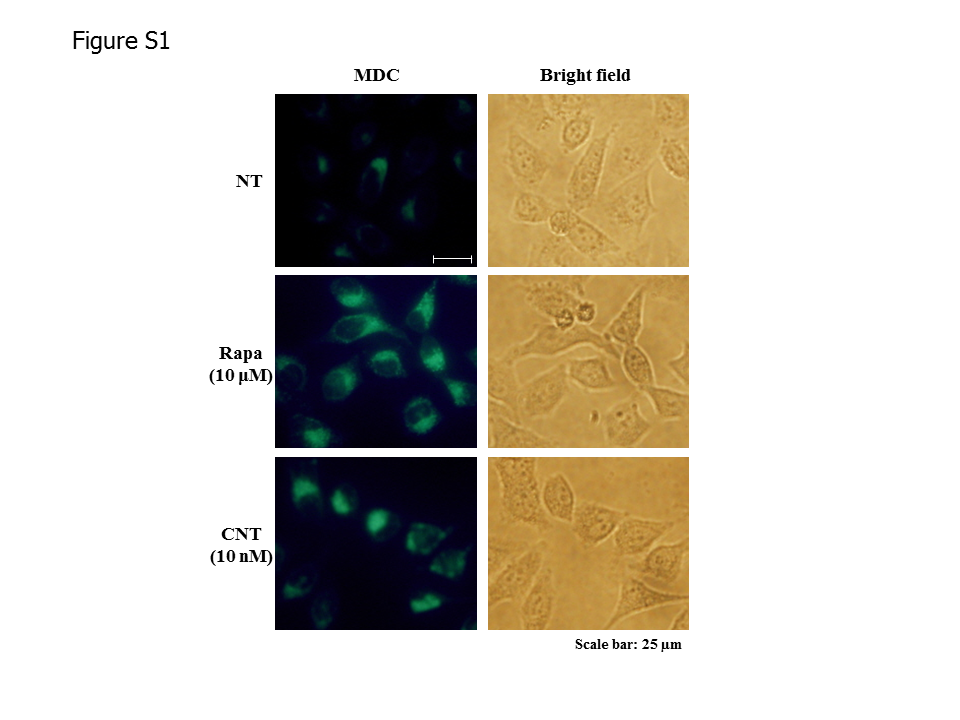

Supplement: Figure S1 — MDC stains autophagosomes in the cytosol but does not stain nuclei in HeLa cells. The vacuoles were monitored under fluorescence field and morphology of cell and nuclei were done under bright field (NT: non-treatment, Rapa: rapamycin, CNT: convallatoxin). Scale bar indicates 25 µm. (TIF) [file pone.0091094.s001.tif]
